# Influence of new coronavirus pandemic on behavior and awareness of young nurses and nursing students in Japan

**DOI:** 10.1186/s12912-021-00724-x

**Published:** 2021-11-24

**Authors:** Mitsuto Hasuike, Yoshiaki Hara, Hiroko-Miyuki Mori, Norio Ideguchi, Fumie Shirai, Yasuko Yoshimura, Ikumi Murakami, Hirohisa Kawahata, Motokuni Aoki, Toshio Ogihara

**Affiliations:** 1 Department of Nursing, Morinomiya University of Medical Sciences, 1-26-16, Nanko-kita, Suminoe-ku, Osaka, 559-8611 Japan; 2 Graduate School of Health Sciences, Morinomiya University of Medical Sciences, 1-26-16, Nanko-kita, Suminoe-ku, Osaka, 559-8611 Japan; 3 Department of Medical Engineering, Morinomiya University of Medical Sciences, 1-26-16, Nanko-kita, Suminoe-ku, Osaka, 559-8611 Japan; 4 Inclusive Medical Science Research Institute, Morinomiya University of Medical Sciences, 1-26-16, Nanko-kita, Suminoe-ku, Osaka, 559-8611 Japan; 5 Department of Acupuncture, Morinomiya University of Medical Sciences, 1-26-16, Nanko-kita, Suminoe-ku, Osaka, 559-8611 Japan; 6 Department of Medical Technology, Morinomiya University of Medical Sciences, 1-26-16, Nanko-kita, Suminoe-ku, Osaka, 559-8611 Japan

**Keywords:** COVID-19, Nurse, Nursing student, Anxiety, Questionnaire survey, Professionalism, Views on life and death

## Abstract

**Background:**

Although mental health disorders of health care workers in the coronavirus disease 2019 (COVID-19) pandemic have been focused, little is known about the psychological impact on nurses and the influence on their behavior and awareness, such as professionalism and views on life and death, in Japan where there are fewer cases of infection and deaths than in other countries. Moreover, the influence of the pandemic on nursing students is still unclear.

**Methods:**

An online questionnaire survey was conducted among nurses and nursing students. Feelings during the state of emergency (at the peak of the pandemic) in Japan, changes in behavior and awareness after the rise of COVID-19, and the associated factors influencing these changes were analyzed, comparing nurses with nursing students.

**Results:**

Significantly increased scores of anxiety/fear (*p* < .005) and voluntary restraint (*p* < .005) and significantly decreased score of motivation (*p* < .005) were observed during the state of emergency in both nurses and students. Scores of experience of discrimination (*p* < .005) and consideration of premature retirement (*p* < .01) were significantly increased in nurses. Moreover, preventive behavior (*p* < .005), lifestyle (*p* < .005), anxiety about nursing (*p* < .005) and views on life and death (*p* < .005) significantly changed after the rise of COVID-19 in both nurses and students. Only nurses reported significant damage to their professionalism (*p* < .01). Anxiety/fear and/or voluntary restraint and/or decreased motivation during the state of emergency were major factors associated with these changes. Also, the type of hospital, experience of care of infected patients and sex affected some of the changes. Voluntary restraint (*p* = .008), increased preventive behavior (*p* = .021) and decreased motivation (*p* = .005) were more marked in nurses than in students, while change in views on life and death was greater in students than in nurses (*p* = .002).

**Conclusion:**

The COVID-19 pandemic has had a psychological impact on nurses and nursing students, associated with changes in behavior and awareness even in Japan. Of note, the COVID-19 pandemic has affected nurses’ professionalism and views on life and death. This study demonstrates the importance of having a coping strategy for anxiety and damaged professionalism in nurses, and education on life and death in nursing students.

**Supplementary Information:**

The online version contains supplementary material available at 10.1186/s12912-021-00724-x.

## Background

A novel pneumonia caused by severe acute respiratory syndrome coronavirus 2 (SARS-CoV-2), which was named coronavirus disease 2019 (COVID-19), emerged in the Chinese city of Wuhan at the end of December 2019, and spread not only domestically in China but also internationally [1]. The World Health Organization (WHO) designated the COVID-19 outbreak a public health emergency of international concern on January 30, 2020 [2] and declared a pandemic on March 11 because of its widespread and rapid rate of transmission [3]. To suppress spread of the virus during the expansion phase of the COVID-19 pandemic, several governments declared a state of emergency and implemented “lockdown” which imposed restrictions on movement, behavior, work and school attendance. Facing this critical situation, health care workers, especially nurses who are directly involved in the treatment of COVID-19 patients and are confronted with the crisis of medical collapse, are at risk of psychological distress, anxiety, fear, alienation, exhaustion and sometimes discrimination [4–10].

In Japan, sporadic outbreaks of COVID-19 began in early March, and the number of infected people rose sharply in late March, mainly in urban areas such as Tokyo. The Japanese government focused on “3Cs” (Closed spaces, Crowded places, Close-contact settings) as infectious environments [11], and strongly encouraged avoidance of the 3Cs in addition to standard infection prevention measures such as wearing a mask, gargling and washing hands. Then, the Japanese government declared a state of emergency on April 16, continuing to May 25, which did not enforce restrictions but requested “voluntary restraint (self-quarantine)” to avoid the 3Cs, different from enforced lockdown in other countries. Despite voluntary restraint not being mandatory and the number of infected patients and deaths being relatively low in Japan as compared to other countries [12], young nurses facing their first experience of an unknown infectious disease must have been under stress, as in other countries. Also, the COVID-19 pandemic would be expected to influence their behavior and awareness such as nursing professionalism and views on life and death. However, little is known about the psychological impact of the pandemic on nurses and changes in their behavior and awareness after the rise of COVID-19 in Japan. Moreover, the influence of the COVID-19 pandemic on nursing students is still unclear.

Here, we investigated the influence of the COVID-19 pandemic on feelings of nurses and nursing students and changes in their behavior and awareness, evaluated the associated factors influencing these changes, and compared nurses with nursing students, employing an online questionnaire survey.

## Methods

### Study design

This was a quantitative study that used an online survey, employing a web-based questionnaire which was generated using Googleform, a cloud-based survey development application, between August 10 and August 24, 2020, when 3 months had passed after the end of the declaration of the state of emergency for the COVID-19 pandemic in Japan. The questionnaire was conducted in 439 nurses who graduated from the Department of Nursing of Morinomiya University of Medical Sciences and 340 current nursing students from the first year to the fourth year at the same university. As of August 10, all subjects were invited to participate in this study, and the due date for questionnaire submission was set as August 24.

### Composition of online questionnaire

Questionnaire used in this study was developed for this study (Additional file 1). The questionnaire consists of attribute data and two major sections, A: Feelings and voluntary restraint behavior during the state of emergency in Japan, and B: Changes in awareness and behavior from before to after the rise of COVID-19 (from 2019 before the rise of COVID-19 to August 2020, which is 3 months after the end of the state of emergency and when participants answered this questionnaire).

Categories and each item of the questionnaire are shown in Table 1. The questionnaire was composed of participants’ demographic characteristics (age, sex, years of experience of nursing, type of hospital, experience of care of patients with COVID-19. and grade for nursing students) and two major sections; A) Feelings and behavior during the state of emergency in Japan, and B) Changes in behavior and awareness from last year before the rise of COVID-19 to August 2020 when 3 months had passed after the end of the state of emergency.

**Table 1:** Categories and items of the online questionnaire survey

Category	Items
Section A: Feelings and behavior during the state of emergency in Japan	
A1: Anxiety/fear about COVID-19	Anxiety about infection with the virus Anxiety about spreading the virus to others Fear of COVID-19 Fear of death from COVID-19
A2: Voluntary restraint	Accepts request for voluntary restraint Maintains self-quarantine Avoids 3Cs Maintains social distancing
A3: Motivation	Positive motivation to care for patients with COVID-19
A4: Experience of discrimination (for nurses only)	Experiences of discrimination against you or your family
A5: Consideration of premature retirement (for nurses only)	Hopes to retire or change job
Section B: Changes in behavior and awareness prior to and after the rise of COVID-19 in Japan	
B1: Frequency of preventive measures	Hand washing Hand sanitization Gargling Use of disposable gloves Cough etiquette Air circulation Wearing a mask
B2: Preventative lifestyle measures	Daily temperature measurement Daily check of physical condition Avoidance of personal outings Avoidance of eating with friends Avoidance of conversations without a mask Awareness of getting enough sleep Awareness of ensuring adequate nutrition Awareness of stress-relieving behaviors Awareness of exercise
B3: Professionalism	Satisfied with your career choice to be a nurse Would recommend the nursing profession to others (For nurses) Find nursing rewarding Motivated to continue in the nursing profession (For nursing students) Motivated to become a nurse
B4: Anxiety about nursing	(For nurses) Anxiety about working as a nurse (For nursing students) Anxiety about future work
B5: Views on life and death	Interest in life Attention to death Time to think about life and death

The online questionnaire survey we used consists of two major sections, A: Feelings and behavior during the state of emergency in Japan, and B: Changes in behavior and awareness and prior to and after the rise of COVID-19 (from 2019 before the rise of COVID-19 to August 2020, which is 3 months after the end of the state of emergency and when participants answered this questionnaire)

Section A of the questionnaire consists of five categories (A1: Anxiety/fear about COVID-19, A2: Voluntary restraint, A3: Motivation, A4: Experience of discrimination and A5: Consideration of premature retirement) for nurses, and three categories (A1, A2, and A3) for nursing students. A1 and A2 consist of multiple items. Each item was scored according to four responses: 1: not at all, 2: not much, 3; a little, and 4: very much

Section B of the questionnaire consists of five categories (B1: Frequency of preventive measures, B2: Preventative lifestyle measures, B3: Professionalism, B4: Anxiety about nursing, and B5: Views on life and death) for nurses and nursing students. All categories consist of multiple items. Each item was scored according to five responses; 1: large decrease, 2: small decrease, 3; no change, 4: small increase, and 5: large increase

### Statistical analysis

For identification of influences of the COVID-19 pandemic on categories of the questionnaire, the mean score of each category was compared to the criterion value, which was a score of 3 in the absence of COVID impact using Weltch’s t-test. Differences between scores of nurses and scores of nursing students were also analyzed using Welch’s t-test.

Multiple regression analysis was employed for evaluation of factors associated with each category in section B of the questionnaire. The independent variables in analysis for nurses were mean score of each category in section A, type of hospital, experience of care of patients with COVID-19 and sex. The independent variables in analysis for nursing students were mean score for each category in section A and sex. SPRC (standardized partial regression coefficient) and 95% CI (confidence interval) of each independent variable were expressed.

All analyses were performed using R (version 4.0.2) [13], and the significance level for each test was set at 0.05.

## Results

### Demographic characteristics

Among the 439 nurses and 340 current nursing students asked to participate, 214 nurses (48.8%) and 320 nursing students (94.1%) responded to the online questionnaire survey, of whom 59 nurses (27.6%) and 5 nursing students (1.4%) were excluded because of missing data, which resulted in a sample of 155 nurses and 315 nursing students (Table 2).

**Table 2:** Demographic characteristics

Nurses (*n* = 155)		Nursing students (*n* = 315)	
Age (y.o.)	24.4 ± 3.06	Age (y.o.)	19.8 ± 1.37
Sex		Sex	
Female	125 (80.6%)	Female	272 (86.3%)
Male	30 (19.4%)	Male	43 (13.7%)
Years of experience	2.11 ± 1.51	Grade	
< 1 year	37 (23.9%)	First year	83 (26.3%)
1 ~ 2 years	36 (23.2%)	Second year	83 (26.3%)
2 ~ 3 years	36 (23.2%)	Third year	83 (26.3%)
4 ~ 7 years	46 (29.7%)	Fourth year	66 (21%)
Working in a hospital that accepts patients with COVID-19	101 (65.2%)		
Experience of care of patients with COVID-19	30 (19.4%)		

The final sample of responding nurses and nursing students were aged 24.4 + 3.1 and 19.8 + 1.4 years respectively, and 125 (80.6%) nurses and 272 (86.3%) nursing students were female. Nurses were at 1 to 7 years after graduation and had 2.11 + 1.51 years of nursing experience. Also, 101 (65.2%) nurses worked in hospitals that accept patients with COVID-19, and 30 (19.4%) nurses had experience of care of patients with COVID-19.

### Feelings and behavior during state of emergency in Japan

**Fig. 1:**
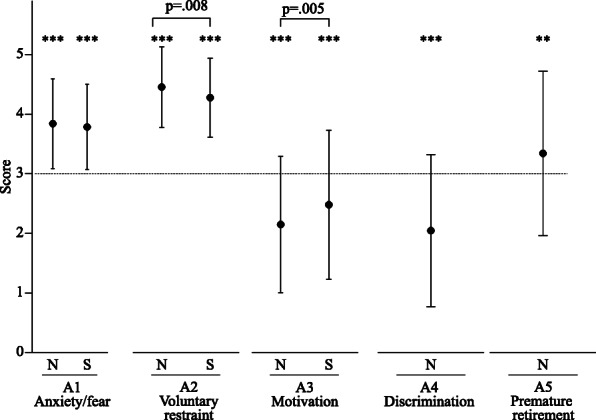
Consciousness and behavior during state of emergency in Japan**.** N; nurses, S; nursing students. A1: Anxiety/fear about COVID-19, A2: Voluntary restraint, A3: Motivation, A4: Experience of discrimination, A5: Consideration of premature retirement. Values are mean ± SD. **: *p* < .01 vs basal level, ***: *p* < .001 vs basal level

Scores of five categories in nurses and three categories in nursing students, which asked about behavior and awareness during the state of emergency in Japan, are shown in Fig. 1. Significantly increased scores of A1 (Anxiety/fear about COVID-19) (*p* < .005) and A2 (Voluntary restraint) (*p* < .005) and significantly decreased score of A3 (Motivation) (*p* < .005), as compared to the basal level, were observed during the state of emergency in both nurses and students. Scores of A4 (Experience of discrimination) (*p* < .005) and A5 (Consideration of premature retirement) (*p* < .01) were also significantly increased in nurses. Although there was no significant difference in A1 score (Anxiety/fear about COVID-19) between nurses and nursing students, A2 score (Voluntary restraint) in nurses was significantly higher than that in nursing students (*p* = .008), and A3 score (Motivation) in nurses was significantly lower than that in nursing students (*p* = .005).

Moreover, as shown in Table 3, nurses who worked in hospitals that accept patients with COVID-19 had significantly higher motivation as compared to nurses who did not (*p* < .05). Also, nurses who had experience of care of patients with COVID-19 felt they experienced discrimination more than nurses who did not (*p* < .01). Concerning sex, a greater drop in motivation was observed in female nurses than in male nurses (*p* < .0001), and female nursing students were more conscious of voluntary restraint than male students (*p* < .05).

**Table 3:** Factors associated with each category of section A

	A1	A2	A3	A4	A5
	Anxiety/fear	Voluntary restraint	Motivation	Discrimination	Premature retirement
**Nurses**					
Independent Variable	SPRC (95% CI)	SPRC (95% CI)	SPRC (95% CI)	SPRC (95% CI)	SPRC (95% CI)
Hospital that accepts patients with COVID-19					
No	–	–	–	–	–
Yes	0.30 (− 0.06 to 0.66)	0.22 (− 0.14 to 0.58)	0.42 (0.08 to 0.76) *	0.34 (− 0.01 to 0.69)	− 0.02 (− 0.39 to 0.34)
Experience of care of patients with COVID-19					
No	–	–	–	–	–
Yes	−0.17 (− 0.59 to 0.26)	0.02 (− 0.41 to 0.44)	−0.06 (− 0.34 to 0.46)	0.57 (0.16 to 0.97) **	−0.30 (− 0.73 to 0.13)
Sex					
Female	–	–	–	–	–
Male	−0.14 (− 0.55 to 0.28)	−0.35 (− 0.76 to 0.06)	0.89 (0.50 to 1.30) ****	0.21 (− 0.19 to 0.60)	0.17 (− 0.25 to 0.58)
**Nursing Students**					
Independent Variable					
Sex					
Female	–	–	–	–	–
Male	− 0.23 (− 0.56 to 0.09)	−0.33 (− 0.65 to − 0.01) *	0.31 (− 0.01 to 0.63)	−0.23 (− 0.56 to 0.09)	−0.33 (− 0.65 to − 0.01) *

*SPRC* Standardized partial regression coefficient, *CI* Confidence interval

*: *p* < .05, **: *p* < .01, ***: *p* < .005, ****: *p* < .001

### Influence of COVID-19 pandemic on behavior and awareness

**Fig. 2:**
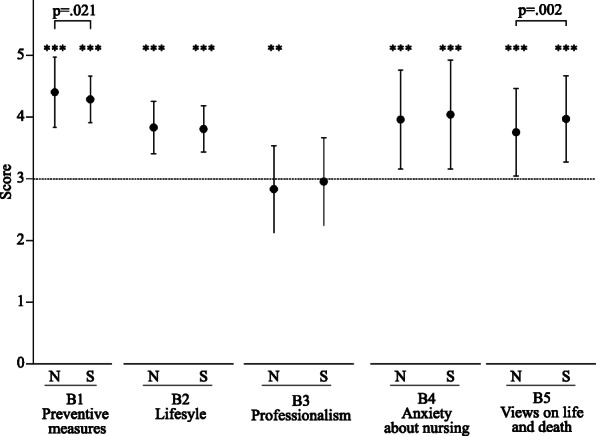
Influence of COVID-19 pandemic on behavior and awareness. N; nurses, S; nursing students. B1: Frequency of preventive measures, B2: Lifestyle to prevent infection, B3: Professionalism, B4: Anxiety about nursing, B5: Views on life and death. Values are mean ± SD. **: *p* < .01 vs basal level, ***: *p* < .001 vs basal level

Fig. 2 shows the scores of five categories in nurses and nursing students, which show changes in behavior and awareness after the rise in COVID-19. In both nurses and nursing students, scores of B1 (Frequency of preventive measures) (*p* < .005), B2 (Lifestyle to prevent infection) (*p* < .005), B4 (Anxiety about nursing) (*p* < .005) and B5 (Views on life and death) (*p* < .005) were significantly increased as compared to the basal levels. B3 score (Professionalism) of nurses was significantly decreased (*p* < .001), while no significant change was observed in nursing students. In addition, B1 score (Frequency of preventive measures) of nurses was significantly higher than that of nursing students (*p* = .021), while B5 score (view on life and death) of nurses was significantly lower than that of nursing students (*p* = .002).

Table 4 shows factors associated with each category of section B. Anxiety/fear about COVID-19 (nurses; *p* < .05, nursing students; *p* < .001) and voluntary restraint (nurses; *p* < .005, nursing students; *p* < .005) during the state of emergency were significantly associated with increase in frequency of preventive measures in both nurses and nursing students. Anxiety/fear about COVID-19 also significantly affected lifestyle in nursing students (*p* < .005), while not in nurses. Voluntary restraint was strongly associated with change in lifestyle in both nurses and nursing students (*p* < .001). Decreased motivation during the state of emergency was associated with damaged professionalism in nurses (*p* < .01) but not in nursing students, and a much greater negative change in professionalism was observed in nurses who considered premature retirement. Anxiety/fear about COVID-19 was also significantly associated with increase in anxiety about nursing (nurses; *p* < .001, nursing students; *p* < .05) and a change in views on life and death (*p* < .001) in both nurses and nursing students. In nurses, experience of care of patients with COVID-19 was associated with change in views on life and death (*p* < .05). Type of hospital did not affect each category. Although there was no significant sex difference in each category in nurses, female nursing students felt anxiety about nursing more than male nursing students (*p* < .05).

**Table 4:** Factors associated with each category of section B

	B1	B2	B3	B4	B5
	Preventive measures	Lifestyle	Professionalism	Anxiety about nursing	Views on life and death
**Nurses**					
Independent Variable	SPRC (95% CI)	SPRC (95% CI)	SPRC (95% CI)	SPRC (95% CI)	SPRC (95% CI)
A1 (Anxiety/fear)	0.22 (0.04 to 0.40)*	0.16 (− 0.02 to 0.34)	0.01 (−0.16 to 0.15)	0.29 (0.12 to 0.46) ****	0.35 (0.16 to 0.54) ****
A2 (Voluntary restraint)	0.29 (0.11 to 0.46)***	0.32 (0.14 to 0.49) ****	0.06 (−0.22 to 0.09)	0.13 (0.03 to 0.36) *	−0.01 (− 0.19 to 0.18)
A3 (Motivation)	0.14 (− 0.02 to 0.29)	0.08 (− 0.08 to 0.24)	0.48 (0.34 to 0.61) ****	−0.05 (− 0.20 to 0.09)	0.10 (− 0.06 to 0.26)
A4 (Discrimination)	0.01 (− 0.15 to 0.17)	0.10 (− 0.06 to 0.26)	0.14 (0.00 to 0.27)	0.13 (− 0.01 to 0.28)	0.11 (− 0.06 to 0.27)
A5 (Retirement)	0.05 (− 0.10 to 0.21)	−0.16 (− 0.31 to − 0.00) *	0.40 (0.26 to 0.53) ****	−0.32 (− 0.46 to − 0.17) ****	−0.02 (− 0.18 to 0.14)
Hospital that accepts patients with COVID-					
No	–	–	–	–	–
Yes	0.08 (− 0.25 to 0.42)	− 0.09 (− 0.42 to 0.25)	−0.16 (− 0.45 to 0.13)	−0.11 (− 0.42 to 0.20)	−0.01 (− 0.35 to 0.34)
Experience of care of patients with COVID-					
No	–	–	–	–	–
Yes	−0.14 (− 0.53 to 0.26)	−0.03 (− 0.42 to 0.37)	−0.12 (− 0.46 to 0.22)	−0.08 (− 0.45 to 0.28)	−0.49 (− 0.89 to − 0.08) *
Sex					
Female	–	–	–	–	–
Male	0.23 (− 0.17 to 0.64)	0.28 (−0.12 to 0.68)	0.12 (− 0.23 to 0.47)	0.15 (− 0.22 to 0.52)	−0.26 (− 0.67 to 0.15)
**Nursing Students**					
Independent Variables					
A1 (Anxiety/fear)	0.28 (0.17 to 0.39)****	0.19 (0.07 to 0.30) ***	0.00 (−0.11 to 0.11)	0.13 (0.01 to 0.24) *	0.26 (0.14 to 0.37) ****
A2 (Voluntary restraint)	0.19 (0.08 to 0.30)***	0.27 (0.16 to 0.38) ****	0.07 (−0.04 to 0.19)	0.06 (−0.06 to 0.18)	0.10 (− 0.01 to 0.22)
A3 (Motivation)	0.02 (−0.09 to 0.12)	0.01 (− 0.10 to 0.11)	0.39 (0.29 to 0.50)****	−0.16 (− 0.27 to − 0.05)**	0.04 (− 0.07 to 0.15)
Sex					
Female	–	–	–	–	–
Male	0.12 (− 0.18 to 0.43)	0.25 (− 0.05 to 0.56)	0.16 (− 0.14 to 0.46)	0.35 (0.03 to 0.67) *	−0.26 (− 0.57 to 0.05)

*SPRC* Standardized partial regression coefficient, *CI* Confidence interval

*: *p* < .05, **: *p* < .01, ***: *p* < .005, ****: *p* < .001

## Discussion

The COVID-19 pandemic has caused a serious public health threat worldwide. In addition to affecting physical health, psychological stress due to fear of the virus and lifestyle restrictions is also a critical issue [14]. In particular, health care workers, including nurses, who come in close contact with infected patients and experience traumatic events such as death, are particularly at risk of stress [15] and are considered a vulnerable group [16]. However, little is known about how much nurses have been psychologically affected by the COVID-19 pandemic in Japan, where there have been fewer infected patients and deaths than in other countries.

First, anxiety/fear about COVID-19 (section A in Table 1) during the state of emergency was significantly elevated among young nurses (Fig. 1), consistent with previous reports [4–10]. Although a significant difference in the severity of psychological symptoms between frontline nurses and second-line nurses has been reported [4], in the present study, the work environment and experience caring for patients with COVID-19 did not affect anxiety/fear about COVID-19. This may be because the number of infected patients and the number of deaths were lower in Japan [12] than in other countries. However, a coping strategy for anxiety in nurses should be established to improve their mental health and prevent burnout or premature retirement. Of note, increased anxiety/fear about COVID-19 among nursing students not in direct contact with patients was the same as on-site nurses (Fig. 1). Similarly, it has been reported that anxiety under a forced lockdown is highly prevalent among nursing students because of economic uncertainty, fear of infection, and social distancing [17]. Our data and a previous report [17] suggest the importance of managing anxiety among nursing students as well as nurses.

Motivation for nursing work during the state of emergency was lower in nurses than in nursing students (Fig. 1), suggesting fatigue and exhaustion among nurses in clinical fields. However, significantly decreased motivation was observed even in nursing students (Fig. 1), suggesting that even Japanese-style voluntary measures, as well as forced restrictions under lockdown, had a psychological effect. However, it is interesting that nurses working in hospitals that accepted patients with COVID-19 had significantly higher motivation than nurses who worked in hospitals that did not (Table 3). It is unfortunate that nurses, especially those who cared for patients with COVID-19, experienced discrimination (Fig. 1b, Table 3). In Japan, discrimination, harassment, hostility, abusive language, and hate speech targeting infected individuals and health care workers were common, especially in the early stages of the pandemic. Although this was probably in the perceived interest of self-protection among the public, it likely accelerated the exhaustion of health care workers and, at times, may have exacerbated stress symptoms among nurses. This may be similar to the hostility towards and discrimination against Asians recorded in a minority of Western countries during the pandemic. Moreover, discrimination, harassment, and even violence against health care workers have been reported in other countries [18–20], causing mental health problems such as stress, anxiety, depressive symptoms, and insomnia [19]. Social efforts to prevent unreasonable attacks on health care workers are urgently needed.

Although the COVID-19 pandemic must have influenced the quality of life of health care workers through its psychological impact, there have been no reports from Japan focusing on changes in behavior and outlook with regard to professionalism and views on life and death. This study evaluated these changes and investigated the associated factors influencing these changes. Concerning behavioral changes, the frequency of preventive measures against transmission was positively impacted (Fig. 2), and greater changes in these factors were observed in nurses and nursing students who were more anxious, fearful, and aware of maintaining voluntary restraint (Table 4). Of note, anxiety/fear about COVID-19 had a stronger impact on nursing students than on nurses (Table 4). These findings are not surprising and suggest that behavioral changes would be forced by stagnation due to anxiety/fear about COVID-19 in nurses, nursing students, and probably the public.

Job satisfaction is considered to be strongly associated with job stress [21, 22], and previous studies have reported decreased job satisfaction among frontline medical staff fighting COVID-19 [23, 24]. In the present study, we focused on job satisfaction and the views of health care workers regarding nursing as a profession. Metrics associated with professionalism, including job satisfaction, were evaluated in four items, as shown in Table 1. Similar to previous reports [23, 24], in the present study, professionalism was significantly negatively impacted in nurses by the rise of COVID-19 (Fig. 2), along with a greater decline in motivation among nurses than nursing students (Fig. 1). Unexpectedly, anxiety/fear about COVID-19, hospital type, and experience caring for patients with COVID-19 did not affect professionalism (Table 4), contrary to a previous report demonstrating an increased level of fear of COVID-19 was associated with job dissatisfaction [24]. Decreased motivation during the state of emergency was strongly associated with the damaged professionalism of nurses in this study (Table 4). Contrary to expectations, our findings showed no difference in the damage to professionalism between nurses working in hospitals that accepted infected patients and those working in hospitals that did not. This suggests a widespread impact on Japanese society caused by this heretofore unknown virus. As damaged professionalism is considered to be associated with premature retirement, burnout, deterioration in the work environment, and patient safety, it is important to build an approach that improves job satisfaction and enhances professionalism in medical fields.

The present study also demonstrated that the COVID-19 pandemic influenced the views on life and death of both nurses and nursing students. As expected, anxiety/fear about COVID-19 among nurses and nursing students and experience caring for patients with COVID-19 were strongly associated with changes in views on life and death. Our data and previous reports [25–27] show the necessity of providing spiritual support for bereaved families as well as health care workers. Of interest, views on life and death varied greatly among nursing students rather than nurses despite the fact that nursing students did not experience traumatic events such as patients’ deaths. This suggests the importance of appropriate education on life and death for young students.

In the present study, there was no major difference in the type of hospital or experience caring for patients with COVID-19 (Tables 3 and 4). In addition, nurses and nursing students showed similar trends, although some differences were observed (Figs. 1 and 2). These results may be unique to Japan, where the number of infected people is low and the medical care system has not yet collapsed. Also, sex differences were found in some categories. Female nurses were less motivated than male nurses during the state of emergency (Table 3), and female nursing students were more likely to maintain voluntary restraint than male nursing students (Table 3). Also, increased anxiety about nursing was higher among female nursing students than among male students. This is consistent with previous reports that showed that women were more likely to have severe symptoms of anxiety and depression [4, 7, 28], experience burnout [10], and practice preventive measures and social distancing [29]. Further study is needed to evaluate sex differences because of the small number of male nurses and male nursing students in this study.

Several limitations of this study should be considered. First, the severity of anxiety and the degree of change in professionalism are not necessarily an accurate assessment because established scales, such as the Generalized Anxiety Disorder scale (GAD-7) [30], the Minnesota Satisfaction Questionnaire (MSQ), Stember’s Web-based 80-question job satisfaction survey [23, 31], were not employed in this study. In addition, this study focused on anxiety specific to COVID-19. As previous reports have evaluated generalized anxiety, it is difficult to compare the reported findings exactly with those of the current study from the perspective of anxiety. Second, the participants were limited to nurses and nursing students in the Osaka area of Japan, where the prevalence of infected patients is higher than in the suburbs and lower than in the Tokyo area. As such, the results of this study cannot necessarily be generalized to all nurses and nursing students in Japan. Third, the questionnaire consisted of questions that did not focus on the current situation, but asked about the subjects’ recollections of past feelings, behavior, and awareness. Bias due to failed recollection cannot be excluded. Fourth, this study did not have a large number of participants. In particular, the percentages of male nurses and nurses who had experience caring for patients with COVID-19 were relatively small. The numbers may not have been sufficient for statistical analysis to evaluate differences in sex and variance among frontline and second-line staff. Finally, the questionnaire used in this study has not yet been validated, and the results of this study may be limited due to a problem with the analysis method based on the assumption that items of the questionnaire can be scored on an interval scale. Further study is required in the future to assess the validity of this questionnaire.

## Conclusions

The COVID-19 pandemic has had a psychological impact, followed by changes in behavior, lifestyle and awareness of nurses and nursing students after the rise of COVID-19, even in Japan where the number of infected patients and deaths, mortality, and severity of COVID-19 are lower than those in other countries and no mandatory lockdown but voluntary restraint was implemented. Especially noteworthy are that the COVID-19 pandemic has influenced the professionalism of nurses and views on life and death of not only nurses but also nursing students. Anxiety/fear about COVID-19, voluntary restraint and decreased motivation at the peak of the pandemic were major associated factors affecting these changes. Our findings will help to understand changes in awareness of nurses and nursing students facing a critical situation, and demonstrate the importance of coping strategies for anxiety and damaged professionalism for nurses and education on life and death for nursing students. These efforts will help improve the clinical environment in the next emerging infectious disease in the future.

## Supplementary Information

**Additional file 1.**

## Data Availability

The datasets supporting the conclusions of this article are included within the article. The datasets used and analyzed during the current study are available from the corresponding author on reasonable request.

## Abbreviations

COVID-19: Coronavirus disease 2019
SD: Standard deviation
SPRC: Standardised partial regression coefficient
CI: Confidence interval
