# Supplementary material for: Influence of new coronavirus pandemic on behavior and awareness of young nurses and nursing students in Japan
Source: BMC Nurs. 2021 Nov 24;20:237. doi: 10.1186/s12912-021-00724-x (PMC8610769; doi:10.1186/s12912-021-00724-x)
Supplement: Supplementary file 1 — Additional file 1. [file 12912_2021_724_MOESM1_ESM.docx]

Questionnaire

**1. Attributes**

I would like to ask you about your personal attributes.

(For nurses and nursing students)

Age

Sex

(For nurses)

Year of graduation

Years of clinical experience

Did the medical institution where you work accept patients with COVID-19?

(yes or no)

Have you cared for patients with COVID-19? (yes or no)

(For nursing students)

Year of study

**2. Section A (Feelings and behavior during the state of emergency in Japan)**

I would like to ask you about your feelings and behavior during the state of emergency. Please recall and answer choosing from the following four options: 1: not at all, 2: not much, 3; a little, or 4: very much.

1) Anxiety/fear about COVID-19

(For nurses and nursing students)

Have you thought that you might be infected with the new coronavirus? (1, 2, 3, 4)

Have you thought that you might transmit the new coronavirus to others? (1, 2, 3, 4)

Did you feel fear concerning infection with the new coronavirus? (1, 2, 3, 4)

Did you think you might die from the new coronavirus? (1, 2, 3, 4)

2) Voluntary restraint

(For nurses and nursing students)

Did you think that the voluntary restraint requested by the government was necessary?

(1, 2, 3, 4)

Were you practicing voluntary restraint? (1, 2, 3, 4)

Were you aware of the 3Cs, and did you take steps to avoid them? (1, 2, 3, 4)

Were you aware of social distancing, and did you act accordingly? (1, 2, 3, 4)

3) Motivation

(For nurses and nursing students)

Did you want to take an active role in caring for patients with COVID-19? (1, 2, 3, 4)

4) Experiences of discrimination

(For nurses only)

Have you or your family felt discriminated against due to your role as a health care worker? (1, 2, 3, 4)

5) Consideration of premature retirement

(For nurses only)

Did you want to quit nursing? (1, 2, 3, 4)

**3. Section B (Changes in behavior and awareness from before to after the rise of COVID-19 in Japan)**

I would like to ask you about the changes in your behavior and awareness. Please recall and answer with the changes from last year before the rise of COVID-19 to now. Please choose from the following five options: 1: large decrease, 2: small decrease, 3; no change, 4: small increase, or 5: large increase.

1) Frequency of preventive measures

(For nurses and nursing students)

Hand washing (1, 2, 3, 4, 5)

Hand sanitization (1, 2, 3, 4, 5)

Gargling (1, 2, 3, 4, 5)

Use of disposable gloves (1, 2, 3, 4, 5)

Cough etiquette (1, 2, 3, 4, 5)

Air circulation (1, 2, 3, 4, 5)

Wearing a mask (1, 2, 3, 4, 5)

2) Preventative lifestyle measures

(For nurses and nursing students)

Daily temperature measurement (1, 2, 3, 4, 5)

Daily check of physical condition (1, 2, 3, 4, 5)

Avoidance of personal outings (1, 2, 3, 4, 5)

Avoidance of eating with friends (1, 2, 3, 4, 5)

Avoidance of conversations without a mask (1, 2, 3, 4, 5)

Awareness of getting enough sleep (1, 2, 3, 4, 5)

Awareness of ensuring adequate nutrition (1, 2, 3, 4, 5)

Awareness of stress-relieving behaviors (1, 2, 3, 4, 5)

Awareness of exercise (1, 2, 3, 4, 5)

3) Professionalism

(For nurses and nursing students)

Satisfied with your career choice to be a nurse (1, 2, 3, 4, 5)

Would recommend the nursing profession to others (1, 2, 3, 4, 5)

(For nurses)

Find nursing rewarding (1, 2, 3, 4, 5)

Motivated to continue in the nursing profession (1, 2, 3, 4, 5)

(For nursing students)

Find study challenging (1, 2, 3, 4, 5)

Motivated to become a nurse (1, 2, 3, 4, 5)

4) Work-related anxiety.

(For nurses)

Anxiety about working as a nurse (1, 2, 3, 4, 5)

(For nursing students)

Anxiety about future work (1, 2, 3, 4, 5)

5) View of life and death.

(For nurses and nursing students)

Interest in life (1, 2, 3, 4, 5)

Attention to death (1, 2, 3, 4, 5)

Time spent thinking about life and death (1, 2, 3, 4, 5)
